# Geospatial analysis for strategic wildlife disease surveillance: African swine fever in South Korea (2019–2021)

**DOI:** 10.1371/journal.pone.0305702

**Published:** 2024-06-21

**Authors:** Satoshi Ito, Jaime Bosch, Cecilia Aguilar-Vega, Hyunkyu Jeong, Jose Manuel Sánchez-Vizcaíno

**Affiliations:** 1 VISAVET Health Surveillance Center, Complutense University of Madrid, Madrid, Spain; 2 Department of Animal Health, Faculty of Veterinary Medicine, Complutense University of Madrid, Madrid, Spain; 3 South Kyushu Livestock Veterinary Medicine Center, Kagoshima University, Kagoshima, Japan; 4 Dodram Pig Research Center, Daejeon, South Korea; University of Illinois Urbana-Champaign College of Veterinary Medicine, UNITED STATES

## Abstract

Since the confirmation of African swine fever (ASF) in South Korea in 2019, its spread, predominantly in wild boars, has been a significant concern. A key factor in this situation is the lack of identification of risk factors by surveillance bias. The unique orography, characterized by high mountains, complicates search efforts, leading to overlooked or delayed case detection and posing risks to the swine industry. Additionally, shared rivers with neighboring country present a continual threat of virus entry. This study employs geospatial analysis and statistical methods to 1) identify areas at high risk of ASF occurrence but possibly under-surveilled, and 2) indicate strategic surveillance points for monitoring the risk of ASF virus entry through water bodies and basin influences. Pearson’s rho test indicated that elevation (rho = -0.908, *p-*value < 0.001) and distance from roads (rho = -0.979, *p*-value < 0.001) may have a significant impact on limiting surveillance activities. A map of potential under-surveilled areas was created considering these results and was validated by a chi-square goodness-of-fit test (X-square = 208.03, df = 1, *p-*value < 0.001). The strong negative correlation (rho = -0.997, *p-*value <0.001) between ASF-positive wild boars and distance from water sources emphasizes that areas surrounding rivers are one of the priority areas for monitoring. The subsequent hydrological analyses provided important points for monitoring the risk of virus entry via water from the neighboring country. This research aims to facilitate early detection and prevent further spread of ASF.

## Introduction

African swine fever (ASF), caused by the ASF virus (ASFV), is one of the most impactful transboundary animal diseases for the livestock industry. In general, pigs are considered susceptible animals, including wild boars. Clinical symptoms vary depending on the balance between the virulence of the ASFV and host immunity; however, clinical stages are mainly classified into four categories: Peracute, Acute, Subacute, and Chronic [1]. Susceptible animals can become infected through contact with infected animals or contaminated materials [2, 3]. Direct transmission via infected animals is deemed important for the spread of the disease over short distances, while indirect contact with contaminated materials may play a major role in disease spread over long distances [4]. An essential aspect of this virus is its high environmental resistance, well known for its ability to remain infectious for long periods under a variety of conditions. The virus is shed in large quantities in the blood where the virus can survive for 15 weeks at room temperature [5]. It can survive for more than three months in raw meat and offal [3, 5], and studies have shown that dry-cured pork, such as Parma ham, remains infectious for 399 days [6]. This unique feature of the virus allows ASF to occur at any location through the movement of ASFV-contaminated material. In endemic countries, long-abandoned infected carcasses increase the chance of exposure to other animals, as well as contaminating the soil, which would lead to further spread of the disease [4, 5].

In Asia, ASF outbreaks have been confirmed in 19 countries (China, Mongolia, Vietnam, Cambodia, North Korea, Laos, Myanmar, the Philippines, South Korea, East Timor, Indonesia, Papua New Guinea, India, Malaysia, Bhutan, Thailand, Nepal, Singapore, and Bangladesh) as of January 22, 2024 [7]. Most of these countries predominantly report ASF from the livestock swine sector. The primary causes are attributed to low biosecurity levels, illegal transportation of infected pigs and/or contaminated pork, or possibly a lack of wildlife surveillance [8]. South Korea is exceptional among these countries in that most notifications have been reported from wild boars [8]. The first ASF outbreak was confirmed in September 2019, at a pig farm in the northwestern part of the country, and the following month the first ASF-infected wild boar carcass was detected near the border with North Korea [9]. Immediately after disease confirmation, fencing around the outbreak area was implemented, and fences kept being built every time the spread of the disease was confirmed. Active searching for wild boars near infected areas and depopulation control are also being promoted. High standards of biosecurity have been established on pig farms to prevent virus entry [10]. Despite these intensive efforts, the disease has continued to spread, with 3549 wild boar cases and 40 outbreaks in pig farms reported by January 22, 2024.

Government-led search teams composed of a variety of people, including civilians, hunters, and military personnel, have been organized nationwide by region to regularly search for wild boars [9, 11]. Furthermore, bounties are offered for the discovery of wild boars to promote the search [11]. However, the terrain of South Korea is largely mountainous and steep, making it challenging to search all areas thoroughly. If there is regional bias in surveillance activities, delays in reporting or missed cases could lead to a silent spread of the disease, which would consequently make disease control even more difficult. Given the reported high density of wild boar populations in South Korea (possibly reaching 10 animals/km^2^) [11], this disease poses a considerable threat not only to the wild boar populations but also to domestic pigs that may potentially come into contact with them.

In addition to the difficulties mentioned above, the constant risk of virus introduction from outside the country should also be considered. Previous studies have raised concerns about the continuous influx of ASFV from North Korea [12], and indeed several ASF-positive wild boar cases have been detected along the country border. In areas facing North Korea, there is a physical barrier called the Demilitarized Zone (DMZ), 248 kilometers long and 4 kilometers wide, extending from the east coast to the west coast, which was established by an armistice agreement in the war between North and South Korea more than 60 years ago [13]. This basically prevents the cross-border movement of animals but several rivers flowing between the two countries could allow infectious agents, such as infected carcasses or parts thereof, to enter the South Korean side. This zone is generally closed to civilians and offers optimal habitat conditions for wildlife, affording a high potential for contact between infectious pathogens and live animals [14]. The importance of rivers and watersheds as priority areas for regular monitoring, besides their role as disease entry routes, is underscored by the finding of Morelle *et al*. [15], that ASF-infected wild boars prefer deathbeds in cool and moist habitats. Indeed, water sites have already been incorporated as part of wild boar search routes in ASF surveillance activities in France [16].

Our analysis focused on the specific timeframe from October 2019 to March 2021. This period aligns with the timeline from the first detection of ASF-positive wild boars in South Korea to the observation of long-jump incidents of wild boars in several regions. These events possibly represent accidental occurrences due to anthropogenic factors, nevertheless, some may also be false long jumps from undetected consecutive occurrences. We combined geospatial analytical methods with statistical approaches to estimating areas at high risk of ASF occurrence but that have been under-surveilled. Hydrological analysis was then applied to indicate strategic surveillance points to monitor the risk of ASF introduction and its area of influence.

## Materials and methods

### Data collection

Epidemiological information on ASF-positive wild boars from 1 October 2019 to 31 March 2021 was obtained from a database provided by PIG PEOPLE, an online newspaper dedicated to swine farming in South Korea [17]. This dataset encompasses details on the finding status, reporting dates, and geographical coordinates of ASF cases.

### Visual understanding of the overall ASF spread trend in the wild boar population

Directional distribution analysis, also known as Standard deviational ellipse analysis [18], was performed to discern the directional trends and spatial extent of ASF cases among wild boars using ArcGIS v10.8.1 software [19]. This technique elucidates the orientation and shape of the spatial expansion of ASF-positive wild boars throughout a defined timeframe. For examining temporal trends in ASF incidents, the study period was segmented into three-month intervals. The country forms and administrative boundaries used for spatial analysis in this study were obtained from the GADM database, freely available for academic use [20].

As a complementary approach to understanding the spatio-temporal trend, we explored the relationship between time elapsed and distance from the first ASF-positive wild boar identified to subsequent cases using the *ggplot2* package [21] within the R programming environment.

### Estimation of under-surveilled areas

**Fig 1:**
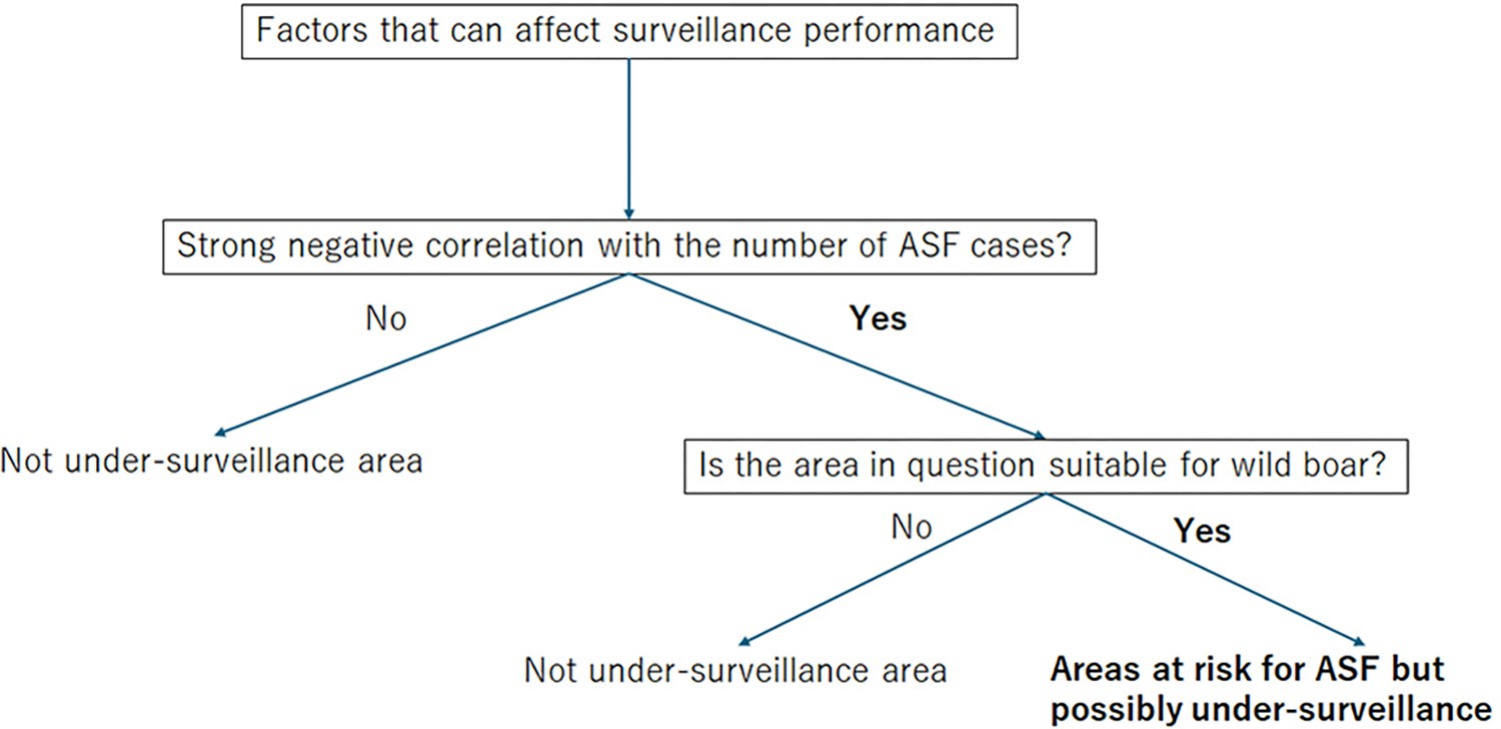
Under-surveilled area estimation flow. The flowchart outlines the criteria used to define under-surveilled areas in this study, along with the methodology that informed this definition.

The potential biases in the spatial distribution of ASF cases, attributable to constrained surveillance measures, were examined through geospatial analytics and statistical methodologies. This methodological framework is illustrated in Fig 1. For the scope of this study, Gangwon Province (Gangwon-do) was selected as the focal area, identified as a region with a widespread presence of ASF as of March 31, 2021. Given that 95.4% of the ASF cases were identified in carcass form, the analysis was conducted irrespective of the state of discovery (i.e., dead, hunted, or trapped).

Here, we selected factors that could affect surveillance performance based on insights from previous wild boar research in Asia [22–24], Korean topographic features [25], and data availability. Five key factors were chosen: elevation, distance from the nearest roads, human population density, percent tree cover (cover tree), and pig density. The rationale was that higher elevations, greater distances from roads, and lower population densities might pose challenges to surveillance activities [25]. Data sources included the SRTM (Shuttle Radar Topographic Mission) 90 m Digital Elevation Model (DEM) Database [26] for elevation, the Korean National Spatial Data Infrastructure Portal [27] for road maps, and the NASA Socioeconomic Data and Applications Center (SEDAC) database [28] for population density. The "Near" tool in ArcGIS 10.8.1 [29] calculated the distance from each ASF case to the nearest road. Information on cover tree was obtained from the U.S. Geological Survey (USGS) database [30] as it may affect the ease of finding wild boars in searching activities. A primary goal in managing ASF among wild boar populations is protecting pig farms from the risk of ASF transmission. Therefore, the vicinity of pig farms ought to guide the prioritization of surveillance efforts. The data on pig density was obtained from the Gridded Livestock of the World (GLW 3) database [31]. To address data not adhering to a normal distribution, Spearman’s rho test was applied to interval scale data, utilizing scales of 50 person/km^2^ for human population density, 50 m for elevation, 5% for cover tree, 20 pig/km^2^ for pig density, and 250 m for proximity to roads (S1 Fig). The association between these factors and ASF case numbers was assessed using the cor.test() function in R, opting for Spearman’s rho as the test method.

To estimate areas potentially overlooked in ASF surveillance, we identified sectors exhibiting a strong negative correlation with ASF incidences that failed to encompass 95% of total case numbers, utilizing the ArcGIS 10.8.1’s “Extract by attributes” tool [32]. Correlation strength was categorized on a scale from very weak to very strong, corresponding to coefficient values of 0.00–0.19, 0.20–0.39, 0.40–0.69, 0.70–0.89, and 0.90–1.00, respectively [33].

Further analysis determined if these areas also constituted optimal wild boar habitats, thereby being classified as “areas at risk of ASF occurrence but possibly under-surveilled”. For this, the Quality of Available Habitat (QAH) map for wild boar by Bosch *et al*., which quantifies habitat suitability across seven levels (from 0 to 2), was employed [34]. The applicability of the QAH map to South Korea was verified by correlating QAH levels with wild boar presence densities, derived from the GBIF database [35], using Spearman’s rho in R. Density was calculated by dividing the number of wild boar presence records by the number of cells for each QAH level. A threshold was established at the QAH level where the number of wild boar presence records exceeded 90% of the total, marking areas as having a high likelihood of wild boar presence. Subsequently, regions satisfying all outlined criteria were delineated with the "Extract by mask" function in ArcGIS 10.8.1 [36].

The validity of the maps created was assessed using a methodology similar to that used in a study by Bosch *et al*. [34]. For the evaluation, we acquired additional data on ASF-positive wild boars from Gangwon-do up until January 13, 2023, obtained from a PIG PEOPLE database [17]. To ascertain the average density of ASF cases within and without the under-surveilled areas, we overlaid the geographic coordinates of reported ASF cases on a map. The density calculation involved dividing the total number of ASF cases by the number of grid cells that covered the mapped area. The expected number of ASF cases for each area was determined by multiplying the average case density across the entire study region by the total number of grid cells within each specific area. The Chi-squared goodness-of-fit test was performed with the chisq.test() function in R to compare the number of reported ASF cases (*n*_reported_) with the number of expected ASF cases (*n*_expected_) in each area. Here, the null hypothesis is "there is no significant difference in the number of cases observed inside and outside of potential under-surveilled areas".

### Identification of strategic surveillance points for monitoring ASF introduction via rivers

We initially applied hydrological analysis in ArcGIS 10.8.1 software to identify secondary water　(which can be interpreted as streams rather than rivers) in South Korea [37]. This was followed by a statistical examination of the relationship between ASF-positive wild boar locations and proximity to water sources. The spatial network analysis of the hydrological system highlighted strategic points for ASF surveillance, focusing on rivers as potential pathways for ASF introduction.

The hydrological analysis was grounded on a DEM derived from SRTM, utilizing a 90-meter resolution. From the hydrological point of view, pixels or cells with values significantly lower in altitude than the neighboring values were equalized, since these would act as a means of escaping the water (sinks). This first step correcting possible errors avoiding sinks in the ground and allowing the water to advance through the pixel and continue to the mouth area as would occur under normal conditions in nature. Upon establishing networks of secondary water streams, we assessed the correlation between ASF-positive wild boar locations and their distances from these water bodies using the Spearman rho test in R. For analytical clarity, distances were grouped by 100-meter intervals and presented in a line graph.

Subsequent steps involved calculating water flow directions to produce a map indicating water movement across the land surface. This was achieved by analyzing the altitude differences between adjacent cells, directing water flow based on the steepest descent, and culminating in flow accumulation analysis. Such analysis quantifies the cumulative water volume converging towards specific points, thereby identifying cells indicative of significant water flow—essentially, rivers and their tributaries. Following the identification of primary rivers and their tributaries or streams, it is essential to construct a comprehensive drainage or river network. This network serves as the foundation for employing Network Analyst tools, facilitating the analysis and management of the hydrological infrastructure.

Identifying drainage or "drain" points within the network constitutes a crucial parameter, marking locations where runoff water transitions into a new hydrological basin. These points delineate significant transitions, where water exits one basin to enter another, serving as strategic locations for hydrological measurements. Following the establishment of the comprehensive network and the integration of its components, spatial analysis was conducted within the network of Korean hydrographic channels, including riverbeds and watercourses. With the Utility Network Analyst tool in ArcGIS, we applied the Trace Downstream command [37] to identify locations where contaminated materials, such as ASF-infected carcasses, could potentially flow from North Korea through the DMZ into South Korea. This process allowed for a holistic assessment of the downstream basin. Modeling of ASFV dispersion pathways within the drainage network took into consideration the convergence points of primary and secondary networks, including rivers, affluents, streams, and channels. Furthermore, we examined intersections or drainage points between basins and river sections, identifying critical water entry and exit points within the basin.

## Results

Epidemiological information from a total of 1,288 ASF cases in wild boar, confirmed by polymerase chain reaction tests in the government laboratory, was obtained. Of these, 812 cases were reported in Gangwon-do by the end of March 2021 (775 were found dead, 23 and 14 were caught by hunting and trapping, respectively).

### Visual understanding of the overall ASF spread trend in the wild boar population

**Fig 2:**
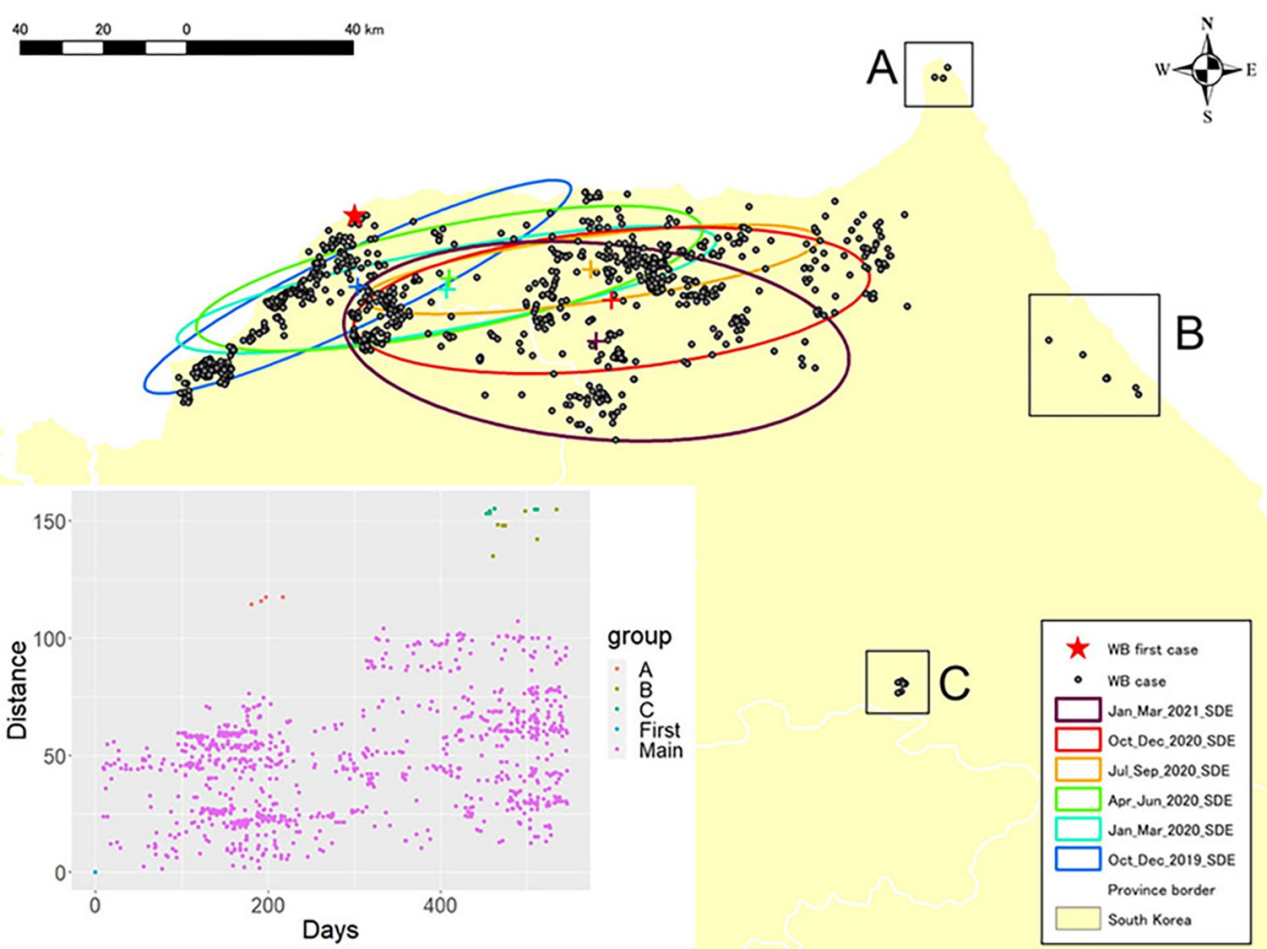
Directional distribution of ASF-positive wild boar and the relationship between time and distance of the ASF event from the initial case in wild boar. Each ellipse indicates the spatial distribution of ASF cases in wild boar over a three-month period (between October 2019 and March 2021). The graduated color of the ellipse represents the time lapsed from earlier (lighter) to more recent (darker). Three isolated groups (named A, B, and C) were observed at a distance from the ellipses. The scatterplot in the lower left shows the association between time elapsed and distance of ASF cases from the initial ASF-positive wild boar.

Directional distribution analysis showed the spatial distribution of ASF-positive wild boar every three months as ellipses (from October 2019 to March 2021). It can be visually understood from Fig 2 that the centroid of the ellipses is gradually moving to the southeast over time, while three groups of ASF-positive wild boar (named groups A, B, and C) were substantially separated from the ellipses. The same trend was also observed from the scatter plots, with a regular trend in the time-distance relationship for ASF expansion, while three irregular groups appeared.

### Estimation of the under-surveilled area

The results of Spearman’s rho test showed that all five selected factors were significantly correlated with the number of ASF cases, with elevation (rho = -0.908, *p-*value < 0.001) and distance from the road (rho = -0.979, *p-*value < 0.001) showing a very strong negative correlation (Table 1). Since 95% of ASF cases were reported below 608 m of elevation or within 3.3 km of a road, areas exceeding either criterion were considered potential under-surveilled areas.

**Table 1:** Results of Spearman’s rank correlation coefficients for the number of ASF cases and each variable.

Variable	ρ (rho)	*p-*value
Human density	-0.612	0.004
Elevation	-0.908	<0.001
Cover tree	0.554	0.011
Pig density	-0.533	0.011
Distance from road	-0.979	<0.001

A strong positive correlation was found between QAH levels and the density of wild boar presence records by QAH levels (rho = 0.786, *p-*value = 0.036), showing the utility of the QAH map as a proxy for the probability of wild boar occurrence in South Korea. Of the total number of wild boar presence records, 93.2% were observed in areas with a QAH level of 1.5 or higher. Thus, areas above QAH level 1.5 were designated as those with a high probability of wild boar presence.

**Fig 3:**
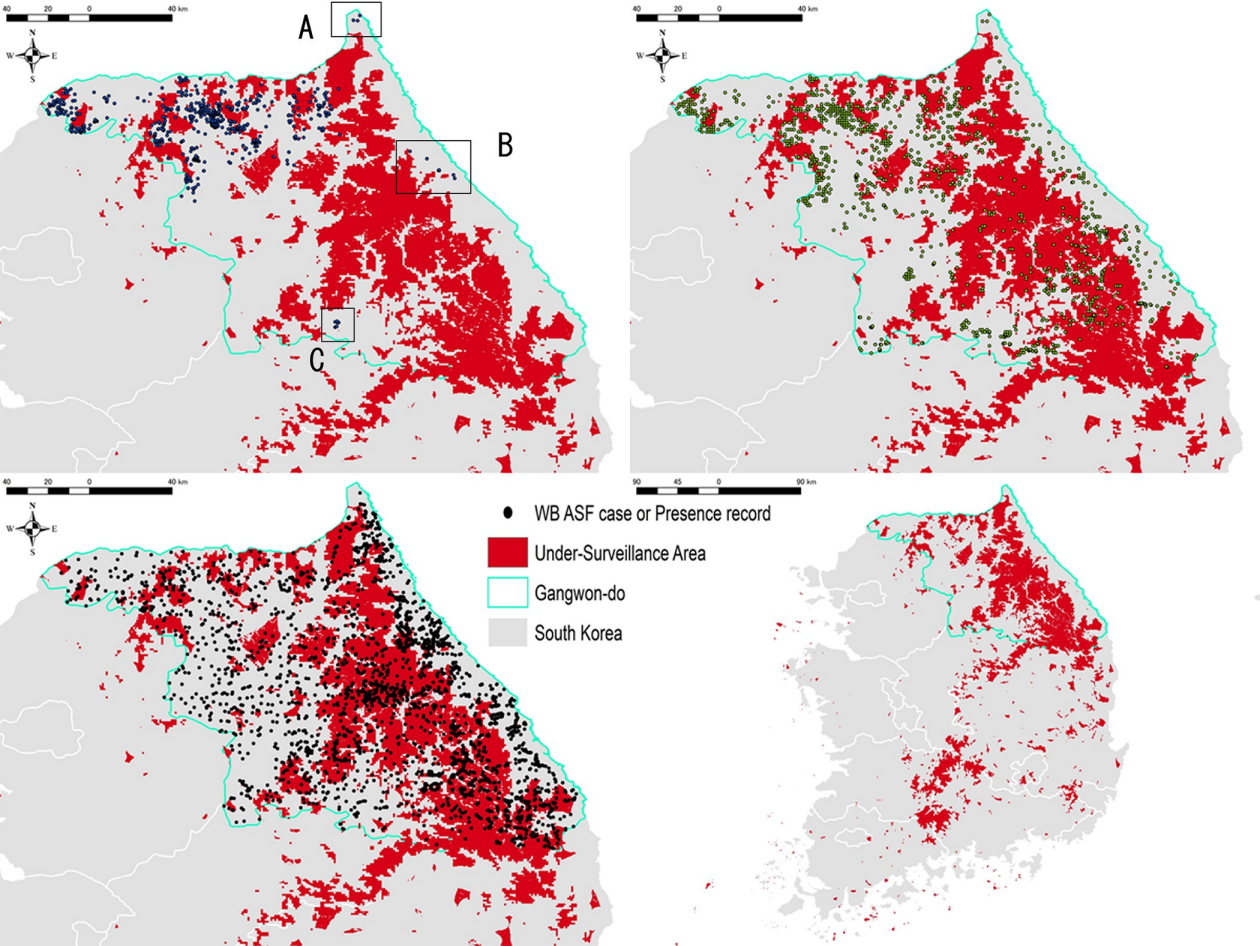
Estimation of the under-surveilled area in Gangwon-do, South Korea. Red-colored areas are those with potential under-surveillance. The upper left and right figures are overlaid with ASF-positive wild boars in Gangwon-do as of 31 March 2021 and 13 January 2023, respectively. The lower left figure is overlaid with wild boar presence records. The lower right figure shows a map of the results obtained in Gangwon-do applied at the national level.

Finally, areas that 1) are at least at an elevation of 608 m or 3.3 km away from roads and 2) have a QAH level greater than 1.5 were designated as areas at high risk of ASF occurrence but potentially under-surveilled (Fig 3).

The Chi-squared test of goodness of fit using epidemiological data of 1857 wild boar cases reported as of January 13, 2023, in Gangwon-do, showed a statistically significant difference between *n*_reported_ and *n*_expected_ in each area (X-squared = 208.03, df = 1, *p-*value < 0.001) (Table 2), thus the null hypothesis was rejected. This indicates that the observed number of reports in the under-surveilled areas is significantly lower than the expected number of reports, demonstrating the validity of the map.

**Table 2:** Results of the Chi-squared test of goodness of fit.

Under-surveilled area	*n*_*reported*_	Normalized density	*n*_*expected*_	Total cell	Residual
No	1442	0.0100	1139.3	144160	9.0
Yes	415	0.0046	717.7	90804	-11.3
Total	1857	0.0079	-	234964	-

### Identification of strategic surveillance points for monitoring ASF introduction via rivers

**Fig 4:**
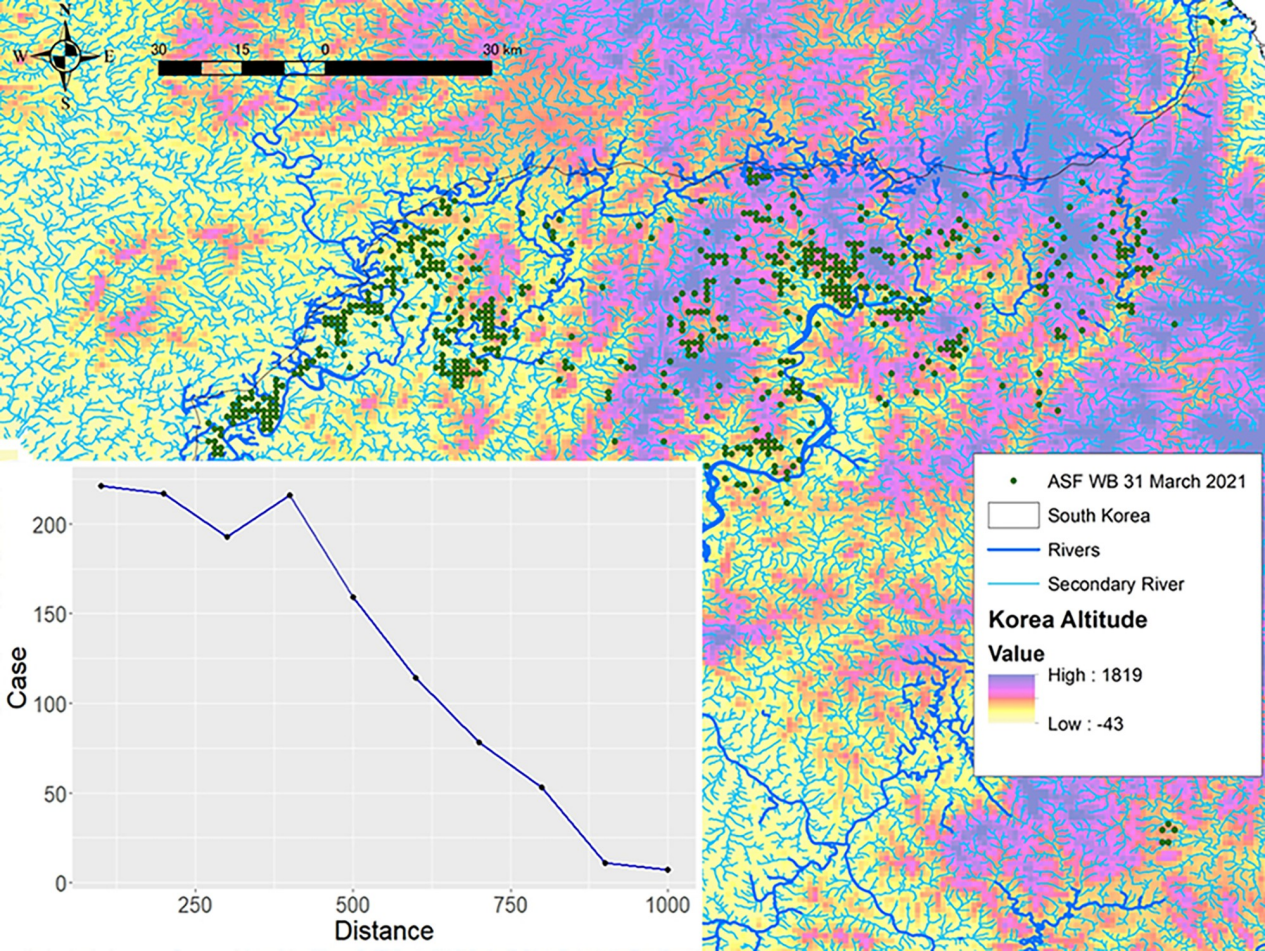
The distance from the ASF-positive wild boar to the nearest water source and spatial network of the hydrological system in South Korea. The line graph on the lower left shows the distance from the ASF-positive wild boar to the nearest water source in 100-meter intervals up to 1000 m. The map shows the developed network of hydrological systems in South Korea overlapping with the elevation map and ASF cases reported as of the end of March 2021.

We developed the Spatial network analysis of the hydrological system (river and streams) to obtain the fluvial dynamics along the network (directions of water flow), and the networks between riverbeds. The Spearman’s rho test showed a very strong negative correlation between ASF-positive wild boar and the distance from the nearest water flow (rivers and streams) (rho = -0.997, *p-*value < 0.001) (Fig 4).

**Fig 5:**
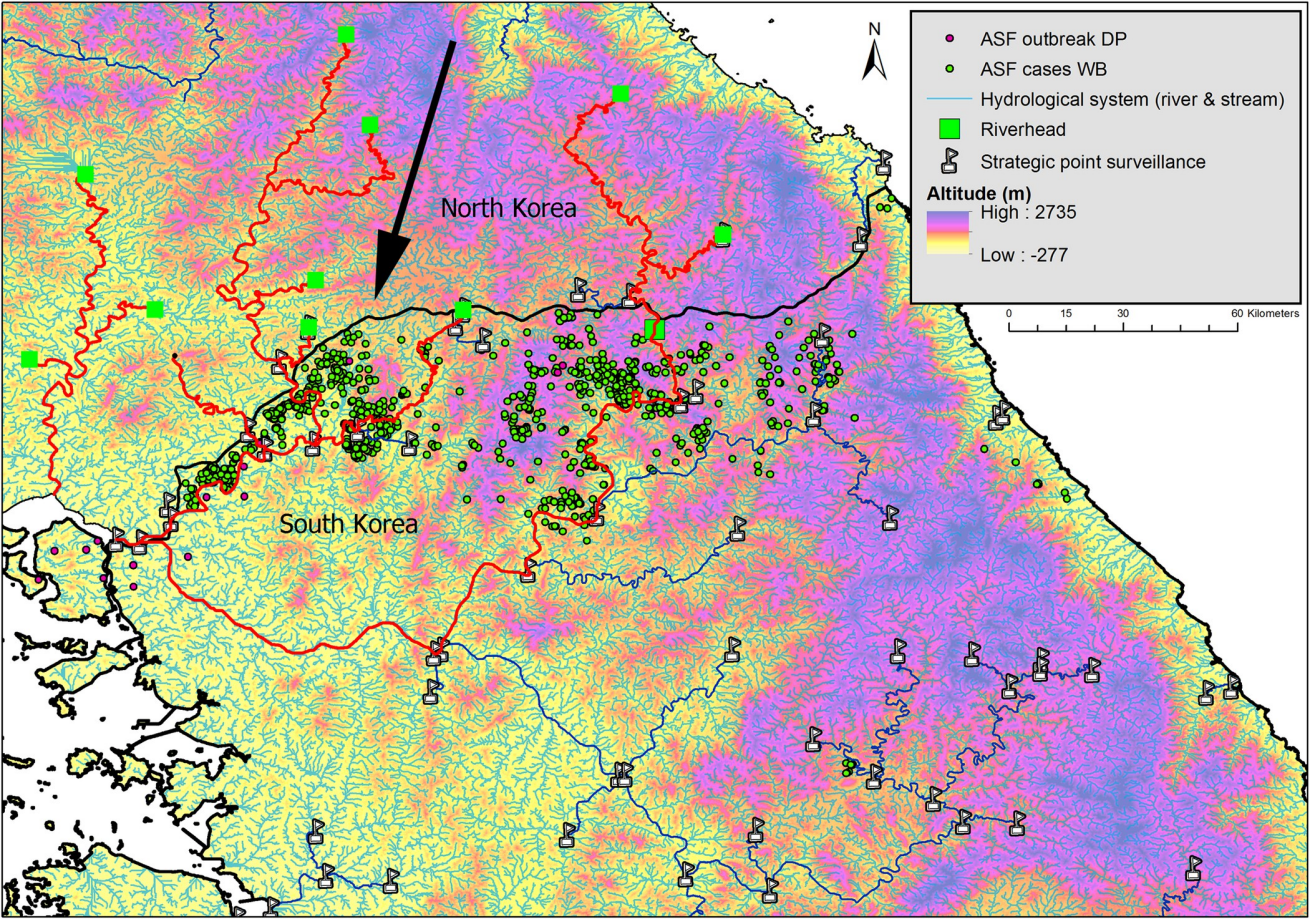
The Korean hydrological system. The red line represents the downstream pathway of the potential risk of ASFV spread from North Korea to South Korea, i.e., the flow of water in the river. The flag symbols represent strategic drainage points for monitoring the risk of ASFV spread through rivers and watersheds. The green square indicates the starting point of the river (Water flow in rivers is from upstream to downstream due to gravity). The black arrow indicates the flow of water along gravity from North Korea through the DMZ to South Korea.

Based on these results, the strategic points (drainage or drain points) for the surveillance of the ASF along the Korean hydrographic basins and influenced areas around streams were estimated and depicted in Fig 5.

The spatial analysis of the Korean hydrographic system, i.e., the downstream flow of the river (under the force of gravity), showed all watercourses that share the border of both countries running from North Korea to South Korea. With the drainage point activated beyond the DMZ, in North Korean terrain, the network assessed the entire affected basin downstream. The direction of water flow from North Korea to South Korea through several rivers and streams crossing the DMZ was indicated.

## Discussion

### Visual understanding of the overall ASF spread trend in wild boar populations

Directional distribution analysis showed a gradual shift in the center of the ellipse to the southeast, presumably due to the direct or indirect contact between wild boars, whereas groups A, B, and C were observed at a distance from the groups in the ellipses. Continuous fencing in infected areas should help limit the movement of wild boars, nevertheless, natural factors such as rivers and steep terrain physically prevent the complete establishment of fences. The wild boar’s ability to swim [38, 39] and dig holes to get through fences may also contribute to the difficulty of disease control. Wild boar can move long distances in a short time, however, the absence of other cases near these isolated groups suggests that other transmission routes may be involved. Anthropogenic factors, such as contaminated clothing, shoes, and vehicles may have carried the virus to distant locations, or the disease appears to have jumped farther due to undiscovered wild boar movements or inaccessible landscape composition.

### Estimation of under-surveilled areas

Prioritization of disease surveillance areas by identifying environmental factors with a high risk of ASF occurrence has been suggested in various investigations, including our previous study [23, 40]. On the other hand, little is known about surveillance bias driven by geographic factors. Great efforts have been devoted to surveillance activities in South Korea, but the complex topography represented by Mt. Taebaek hinders an even and extensive monitoring effort.

The obtained results showed that areas with an elevation greater than 608.0 m or further than 3.3 km from the road plus a QAH level of greater than 1.5 were “areas at high risk of ASF occurrence but possibly under-surveilled”. Here, significant correlations were observed between all five selected factors and the number of ASF cases. Elevation and distance from roads showed very strong negative correlations, raising the possibility that reports are biased in areas with poor access, as expected. Human population and pig densities showed moderate negative correlations, while cover trees, contrary to expectations, showed a positive correlation. The cover tree probably reflects the tendency of wild boars to inhabit forests. The average elevation of Gangwon-do is approximately 508.4 meters, ranging from sea level to 1540 meters. Most of the region, about 73.7%, lies below 600 meters. Consistent with this, about 84.8% of wild boar cases have been reported at elevations of 508 meters or lower, suggesting that most cases occur in locations that are more accessible. However, wild boars can be found at elevations exceeding 2,000 meters [7, 41], indicating that infections can spread even in rugged mountainous areas. While steep terrain poses significant challenges to consistent human monitoring activities, it simultaneously offers convenient hideouts for wild boars, potentially complicating efforts to control the spread of disease.

The under-surveilled map was developed based on data from ASF cases reported in Gangwon-do up to 31 March 2021, but overlaying ASF cases up to 13 January 13, 2023, shows that there are still many under-surveilled areas remaining. The use of drones and the introduction of detection dogs since fall 2022 have contributed to improving the bias of the surveillance area [42–44], however, searching over a wide area remains a challenge. In contrast to European ASF infection areas, which are primarily composed of flatlands and gentle hills, the control of wildlife is even more challenging in Asia due to its predominantly steep and complex terrain. In Japan, where Classical swine Fever is spreading nationwide, the rugged terrain provides hiding places for wild boars, presenting a major challenge for disease management [22, 45]. Given this context, the isolated A, B, and C groups in Fig 3 are surrounded by under-surveilled areas, which may have resulted in the appearance of jumps in cases. Previous studies have shown that notifications at distances of 30–35 km from others can be considered anthropogenic [46], and group C, which is 80 km away from the nearest case, is possibly a geographical leap introduced by humans.

The Korean government has devoted considerable effort to the search for wild boars, conducting intensive searches around fenced ASF-affected areas and requiring that any carcasses found be reported to local authorities. Nonetheless, the problem has been raised of people who do not have sufficient biosecurity knowledge joining surveillance activity teams, thus resulting in their unintentional contribution to the spread of infection. The Civilian Control Zone, a 5–10 km wide area outside the DMZ, is restricted to civilians but allows for the stationing of military troops and land use for agriculture. Given that many ASF-positive wild boars have been found in this area, visitors could possibly carry the virus outside. Such inappropriate human behavior has been reported in European ASF outbreak areas, where it is considered one of the high-risk behaviors [11, 47].

### Identification of strategic surveillance points for monitoring ASF introduction via rivers

The relationship between water sources and wild boars has been explored in various studies across Central and Eastern Europe, in addition to the research conducted by Morelle *et al*., [15]. In the Czech Republic, findings revealed that 59.6% of ASFV-positive and 76.2% of ASFV-negative wild boar carcasses were located within 100 meters of a water source, with nearly all carcasses discovered within a 500-meter radius of water sources [48]. Similarly, research in the Baltic regions indicated a link between the likelihood of identifying at least one ASF-positive wild boar case and the proximity to wetlands and water bodies [49]. Romania also recognizes waterways as crucial risk factors, emphasizing concerns regarding water supply for backyard animals and the potential for waterway contamination by wild boar carcasses and waste [50]. Notably, in South Korea, the Yeoncheon Imjin River was reported to have turned red with pig blood following the culling of pigs in an infected zone [11].

The hydrological analysis was applied to understand the correlation between ASF-positive wild boar incidences and their proximity to water sources. The findings underscored a significant negative correlation, with 80% of ASF-positive cases occurring within 500 meters of flowing water (rivers and streams). Given that 95% of ASF-positive wild boars in South Korea were discovered as carcasses, the proximity to water sources significantly increases the likelihood of encountering ASF-positive carcasses, consistent with earlier studies [15, 48]. The high resistance of ASFV to diverse environmental conditions [4, 5] implies that uncollected infected carcasses not only heighten the risk of virus contamination in the surrounding soil but also facilitate potential direct or indirect interactions among wild boars [5, 51]. Extreme weather events, such as heavy rains and typhoons in summer, potentially sweep the carcasses into rivers, thus amplifying the threat of ASF spread to new regions. Consequently, riverine environments should be emphasized for ASFV risk management and continuous surveillance. Fig 5 shows several rivers that flow between the two countries on the Korean Peninsula. While the movement of wild boar is restricted by a solid fence separating the two nations, the risk of infectious materials being carried into South Korea via large rivers cannot be ignored. For example, the region to which isolated Group A belongs borders North Korea and rivers flow nearby.

The drainage points affected between basins and sections of rivers were estimated in the outbreak areas. Identifying these strategic surveillance locations can help in monitoring the ASF spread through South Korean river basins. Previous studies suggest that the average wild boar population density in the examined area exceeds 8–10 animals/km^2^ [52, 53], notably high compared with ASF-infected regions in Europe [54]. The repeated ASF occurrence in the same areas suggests the possibility of high virus contamination in the environment. Supporting this, the government has reported the detection of ASFV genes in various environmental samples from the ASF-infected area [55]. In light of this, the strategic points (drainage points) that we have established for ASF surveillance along the Korean hydrographic basins and surrounding areas could serve as one of the most effective tools for monitoring and controlling the disease in impacted regions, as well as enhancing the early detection of the disease in areas not yet affected.

### Challenges facing this study

A limitation of this research was its reliance solely on positive case data, due to a lack of available data on ASF-negative wild boars. Consequently, the identification of potential areas vulnerable to surveillance bias was confined to Gangwon-do, a region where cases have been widely reported. It is important to acknowledge that the factors considered in assessing surveillance capability were chosen based on available data, leading to a constrained analysis. Addressing these data gaps would enable a more comprehensive evaluation and help identify vulnerabilities in potential future outbreak zones. Those familiar with the context in South Korea will likely find the results of this analysis intuitively understandable. Effective data visualization can enhance mutual understanding among stakeholders, thereby encouraging collaborative efforts.

This study highlighted aspects of surveillance for ASF control that could be improved based on geographical factors, including waterways. Beyond these, it is crucial to monitor additional routes for the potential introduction and spread of ASF. The presence of ASFV in imported livestock products [56] underscores the need for rigorous, ongoing surveillance of international human and goods movement across all ports. The role of scavengers in the spread of the disease is currently a subject of debate among researchers [57, 58], with their impact yet to be documented in South Korea [11]. Nonetheless, investigating species like *Aegypous monachus* and other carrion birds that migrate from ASF-affected neighboring countries is essential for managing transmission risks.

## Conclusion

A primary objective of this study was to elucidate the mechanisms underlying the expansion of ASF and identify key risk factors by focusing on the initial stages of the epidemic, where the spread was most prominent. This approach aimed to contribute to the development of strategic surveillance systems for wildlife disease control. Geospatial analysis revealed that, beyond the movement of wild boars, various elements—ranging from proximity to neighboring countries to long-distance viral dispersal prompted by human activities, and the geographical biases in surveillance efforts——likely played roles in disease spread.

The Under-surveilled map, statistically analyzed to include cases reported up to January 13, 2023, has thus demonstrated its current validity, indicating that geographic challenges persist as substantial hurdles in disease containment strategies. The hydrological analysis has further highlighted riverine regions as priority areas for ASF vigilance, identifying essential locations for monitoring potential viral entries through water pathways from the neighboring country.

Most countries that have confirmed ASF in wild boar populations are still suffering from the epidemic. The findings of this study underscore its importance not only for South Korea but also for other countries not yet infected or in the early stages of the epidemic.

## Supporting information

S1 Fig Histogram of ASF cases in wild boar for each selected factor. Interval scales of 50 person/km^2^, 50m, 5%, 20pig/km^2^, and 250m were applied for human population density, elevation, cover tree, pig density, and distance from road, respectively. The results of Spearman’s rho test were listed above the histogram.
(TIF)
