# Supplementary figures and images for: Geospatial analysis for strategic wildlife disease surveillance: African swine fever in South Korea (2019–2021)
Source: PLoS One. 2024 Jun 21;19(6):e0305702. doi: 10.1371/journal.pone.0305702 (PMC11192348; doi:10.1371/journal.pone.0305702)

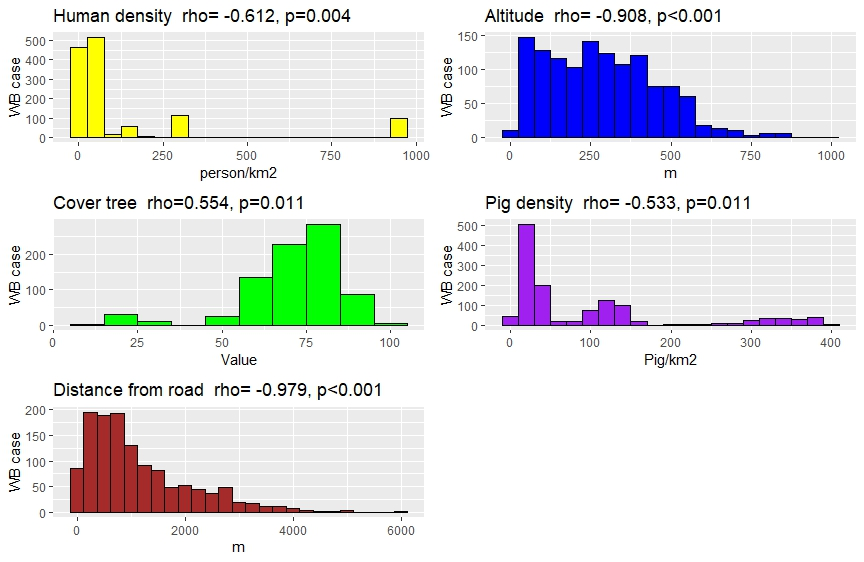

Supplement: S1 Fig — Interval scales of 50 person/km2, 50m, 5%, 20pig/km2, and 250m were applied for human population density, elevation, cover tree, pig density, and distance from road, respectively. The results of Spearman’s rho test were listed above the histogram. (TIF) [file pone.0305702.s001.tif]
